# Role of advanced glycation end products on vascular smooth muscle cells under diabetic atherosclerosis

**DOI:** 10.3389/fendo.2022.983723

**Published:** 2022-08-31

**Authors:** Lin Mao, Ruili Yin, Longyan Yang, Dong Zhao

**Affiliations:** Beijing Key Laboratory of Diabetes Prevention and Research, Center for Endocrine Metabolic and Immune Diseases, Beijing Luhe Hospital, Capital Medical University, Beijing, China

**Keywords:** atherosclerosis, diabetes mellitus, advanced glycation end products, vascular smooth muscle cells, receptor for advanced glycation end products

## Abstract

Atherosclerosis (AS) is a chronic inflammatory disease and leading cause of cardiovascular diseases. The progression of AS is a multi-step process leading to high morbidity and mortality. Hyperglycemia, dyslipidemia, advanced glycation end products (AGEs), inflammation and insulin resistance which strictly involved in diabetes are closely related to the pathogenesis of AS. A growing number of studies have linked AGEs to AS. As one of the risk factors of cardiac metabolic diseases, dysfunction of VSMCs plays an important role in AS pathogenesis. AGEs are increased in diabetes, participate in the occurrence and progression of AS through multiple molecular mechanisms of vascular cell injury. As the main functional cells of vascular, vascular smooth muscle cells (VSMCs) play different roles in each stage of atherosclerotic lesions. The interaction between AGEs and receptor for AGEs (RAGE) accelerates AS by affecting the proliferation and migration of VSMCs. In addition, increasing researches have reported that AGEs promote osteogenic transformation and macrophage-like transformation of VSMCs, and affect the progression of AS through other aspects such as autophagy and cell cycle. In this review, we summarize the effect of AGEs on VSMCs in atherosclerotic plaque development and progression. We also discuss the AGEs that link AS and diabetes mellitus, including oxidative stress, inflammation, RAGE ligands, small noncoding RNAs.

## Introduction

With the development of global economy and production technology, there were significant changes in human dietary structure and lifestyle, which have aggravated the global burden of cardiovascular disease (1, 2). Atherosclerosis (AS) is a chronic inflammatory disease, which is a major cause of cardiovascular events (3). The risk factors causing AS are divided into physiological and pathological risk factors, the former includes unhealthy dietary intake, lack of physical activity, genetics, specific population (age, race, nationality and gender) and smoking, the latter includes hyperlipidemia, pre-diabetes/diabetes, hypertension, obesity and renal dysfunction (4). AS is the most common type of disabling and fatal disease among the complications of diabetic macrovascular disease (5). Dysfunction of lipid metabolism accompanying systemic metabolic diseases in AS is an important risk factor and characteristic (6). Hyperglycemia, dyslipidemia, advanced glycation end products (AGEs), inflammation and insulin resistance, which strictly involved in diabetes, are closely related to the pathogenesis of AS (7, 8). Compared with non-diabetic patients, diabetic patients have a 2-5 fold increased risk of developing AS, the degree of plaque lesions is severe which often involves multivessel disease and is characterized by extensity, acceleration, vulnerability (9, 10). Sustained high-glucose environment provides sufficient reaction substrates and increases the oxidation of the microenvironment of vascular wall, aggravating vascular wall damage and atherosclerotic plaque vulnerability (11). In addition to lipid content, atherosclerotic plaques include different cell types, including vascular smooth muscle cells (VSMCs) and inflammatory cells (such as macrophages, dendritic cells, and lymphocytes), extracellular matrix (ECM) proteins, and cell fragments (12).

VSMCs are important participant in early and late-stage AS (13). VSMCs are exquisitely sensitive to stimulation, and responsible for contraction and relaxation in physiological state, thus regulating intravascular blood flow (14). After endothelial cell dysfunction, a variety of bioactive mediators released from the injured site induce phenotype change of VSMCs from the quiescent “contractile” phenotype state to the active “synthetic” state, which is the key segment affecting the progression of AS (15–18). Diabetes, a disease characterized by persistent high glucose status, continuously induces the production of reactive oxygen species (ROS), breaks the redox balance, induces inflammatory response and phenotypic transformation, leads to vascular remodeling (19, 20). Many studies have reported that long term glycemic control is beneficial to the development and progression of diabetic cardiovascular complications (21–24).

Recent studies suggest that AS is related to the “metabolic memory” theory marked by AGEs, which means that even blood glucose concentration in the normal range, the vascular events risk is amplified (25, 26). AGEs are a class of complexes produced by non-enzymatic reactions of glucose and derivatives, which exacerbate AS in a direct or indirect manner, direct manner means the capture and cross-linking of proteins, and indirect manner by binding to specific receptors on the cell surface (27). Binding of specific receptor for advanced glycation end-product (RAGE) and AGEs carries a big weight in the function of atherosclerotic VSMCs. RAGE expressed on VSMCs surface and mediates multiple damage mechanisms, thereby affecting the progression of AS (28–30). AGEs also have adverse effects on erectile dysfunction (ED), a risk factor for cardiovascular disease in diabetes, and diabetes retinopathy (31, 32). Decreased NO and increased prethrombotic factors result in endothelial dysfunction and thrombosis, while an increase in nuclear factor kappaB (NF-κB) with inflammatory reaction (33, 34). AGEs induce apoptosis of retinal pericytes, differentiation of osteoblasts and calcification, and exert toxic effects on retinal capillaries (35). In this review, we aimed to explore the effects of AGEs on VSMCs function in AS and discuss the AGEs that link AS and diabetes mellitus, including oxidative stress, inflammation, RAGE ligands, small noncoding RNAs and other aspects.

## VSMCs in different stages of AS

As the main functional cells of vascular, VSMCs play different roles in each stage of atherosclerotic lesions. Abnormal proliferation of VSMCs is a common and important mechanism involved in the pathogenesis of many vascular diseases, including AS and aortic aneurysm formation (36).

AS can be divided into pre-atherosclerotic and atherosclerotic stage, in which the atherosclerotic stage can be subdivided into early AS, late AS and clinical sequelae. The prominent features of pre-atherosclerotic stage are diffuse intimal thickenings (DITs) and intimal lipid streaks, in which DITs considered to be the precursor of AS (37, 38). Different from the normal VSMCs in the media, VSMCs in the thickened intima showed a synthetic phenotype with low contractile protein expression and high extracellular matrix (ECM) expression (39, 40). At this stage, VSMCs slightly decreased the content of cholesterol esterase and ATP binding cassette transporter (ABCA1) (41, 42), which led to the production of VSMCs-derived foam cells (43), and established the pathological basis for the subsequent AS progress. In the early stage of AS, pathological intimal thickenings (PITs) occur, which are manifested intimal deep lipid pools formation on the basis of abundant VSMCs and ECM (12, 38). Complex pathological processes such as lipid accumulation, inflammatory stimulation and phenotype transformation of VSMCs promote DITs to PITs. Studies have confirmed that VSMCs are the main source of ECM that plays a central role in initiation of AS (44–46). The development from DITs to PITs is accompanied by loss of VSMCs marker α-smooth muscle actin (α-SMA), which may be the result of phenotypic transformation (47, 48) or death (49, 50). Macrophages can be absent in early PITs, and are essential to the progression of late PITs to fibroatheromas (12). The co-expression of α-SMA and CD68 cells in human atherosclerotic plaques suggesting that VSMCs also play an important role in the cells expressing macrophage markers (51, 52). In addition, there is an interaction between macrophages and VSMCs (53). VSMCs secrete diversified active factors to recruit macrophages to form PITs (54–56), and macrophages have an effect on VSMCs proliferation, migration and phenotypic transformation (57, 58). The next stage of PITs is fibroatheromas, in which fibrous caps as well as necrotic cores appear, which originate from the deep intima lipid pools as well as VSMCs and macrophages with insufficient cell clearance (59, 60). Macrophages phagocytize deposited lipids to become foam cells (61), after exceeding the scavenging capacity of macrophages, they resulted in sustained inflammatory response, caused the death of macrophages- and VSMCs-derived foam cells, then secondary necrosis (62, 63). Fibrous cap is a healing reaction accompanied by injury, proliferation and migration of medial VSMCs stimulated by a variety of materials (47, 64, 65). In addition, VSMCs has long been considered to contribute to calcification. In early fibroatheromas, the causes of calcification include calcified microvesicles from macrophages and VSMCs (66, 67), release of apoptotic bodies and activity of osteochondrogenic cells (68, 69). In the final phases, The main characteristics are increased cholesterol and calcification in necrotic core and MMPs related fiber cap decomposition and remodeling (70). Both VSMCs death in the fibrous cap and necrotic core enlargement will lead to fibrous cap thinner, which makes the plaque easier to rupture (71, 72). Plaque rupture is conversely correlated with VSMCs, and the proliferation, migration and death of VSMCs are important segments (73). The role of VSMCs in different stages of AS was summarized in **Table 1**.

**Table:** Table 1 VSMCs in different stages of AS.

Stage	Subject	Features	Effects
**DITs**	Human	Decreased contractile genes and increased ECM components	Synthetic phenotype VSMCs in part through decreased expression of cholesterol esterase and reduced cholesterol efflux transporter ABCA, resulting in increased tendency towards foam cell formation.
**PITs**	Human	The formation of an extra-cellular lipid pools deep in the intima, underlying and abundant VSMCs and ECM	VSMCs generate lipid retentive ECM to promote the formation of VSMCs-derived foam cells and recruit monocytes.
**Late AS**	Human	The presence of a fibrous cap and a necrotic core	1)VSMCs contribute to the majority of plaque cell phenotypes and promote the development of necrotic core and inflammation.
			2)VSMCs promote calcification.
**Clinical sequelae**	Human	Plaque rupture or erosion	VSMCs show little proliferation, but increased death, through apoptosis and necrosis.

## Mechanisms of VSMCs in AS

As is a chronic inflammatory disease, which is closely related to VSMCs function. VSMCs mainly affect the progression of AS through proliferation, migration and phenotypic transformation. In VSMCs, TNF-α induce nuclear translocation of p65 and STAT3, which may be associated with TNF-α-regulated target promoters, such as monocyte chemoattractant protein-1 (MCP-1) and intercellular cell adhesion molecule (ICAM-1) (74). It is reported that 17β-estradiol (E2) downregulate of tumor-necrosis-factor-related apoptosis-inducing ligand (TRAIL) expression *via* suppression of NF-κB, thereby inhibiting rat VSMCs proliferation and migration (75). Systemic knockout of IL-1β or IL-1 receptor type 1 (IL-1R1) reduced plaque formation, whereas knockout of the IL-1 receptor 1 antagonist gene (IL-1Ra) increased plaque development. Furthermore, treatment with IL-1β neutralizing antibody western diet feeding to apoE ^-/-^ mice reduces overall plaque burden (76–79). However, there are currently no preclinical studies showing benefits of inhibiting IL-1β in advanced atherosclerotic stage.

Recently the effect of noncoding RNAs on regulating AS has drawn attention. Continued improvements in molecular sequencing technologies have led to a greater understanding of AS at the single-cell, chromatin, and epigenetic levels. Circular RNAs (circRNAs) are endogenous regulatory RNAs, which are covalently closed loops after reverse splicing due to the lack of 3’-poly-a tail and 5’- cap (80). Studies have demonstrated that most circRNAs regulate gene expression post-transcriptionally through sponging microRNAs (miRNAs), for example circACTA2, circ-SATB2, circDiaph3, circ_0020397, circTET3, circCCDC66, and miR-541, miR-195, miR-146a, miR-133, miR-214, miR-34a (81, 82). It is suggested that circRNAs and miRNAs may be a new target spot and a new hot point in preventing dysfunctional VSMCs in AS. Connective tissue growth factor (CTGF) plays a crucial role in the VSMCs proliferation and migration to response stimulation of by hyperglycemia (83, 84), AGEs (85), and hypoxia (86). Silence of CTGF increases VSMCs proliferation time by prolonging cell G0/G1 phase, blocking VSMCs into S phase (85). VSMCs proliferation was associated with increased expression of RAGE and its ligands. High mobility group box-1 (HMGB1) plays a pivotal role after vascular injuries. Human VSMCs were treated with HMGB1 (100 ng/ml), markedly increased osteoprotegerin production which mediated by activator protein 1(AP-1), as well as also affected the migration ability of VSMCs (87, 88). Knockdown of S100B by shRNA inhibits PDGF-BB-induced VSMCs proliferation and migration *in vitro* (89). The phenotypic transformation of VSMCs is also a key mechanism in AS progress. Recent studies have reported that Nidogen-2 and MEF2B play an important role for maintenance of VSMCs identity. Nidogen-2 is a basement membrane glycoprotein that enhances the interaction between Jagged1 and Notch3 and subsequent Notch3 activation. Compared with wild-type mice, Jagged1 overexpression attenuated the inhibition of neointima formation in nidogen-2^-/-^ mice (90). Cyclic stretch enhances Nox1 mediated ROS production through MEF2B activation signal and causes VSMCs to switch to a synthetic phenotype (91). Atherosclerotic plaque and vascular calcification constitute two features of AS. MFN2 and LncRNA H19 have been shown to promote calcification of VSMCs (92, 93). The stability of later period of atherosclerotic plaque was dominated by FAM172a, RAGE, galectin-3, autophagy and apoptosis (94–97). Trans-differentiation of VSMCs into a macrophage-like state to phagocytize lipids and form more foam cells are both an interfering factor affecting plaque stability (30, 97). The above mechanisms of VSMCs in AS were summarized in **Table 2**.

**Table:** Table 2 Mechanisms of VSMCs in AS.

Function	Subject	Treatment	Effect
**Proliferation**	Rat	TNF-α	TNF-α stimulation induced p65 and STAT3 phosphorylation and promoted translocation of these molecules into the nucleus, activating NF-κB and proinflammatory gene
	Rat	TNF-α	E2 inhibits VSMCs proliferation and migration by downregulation of TRAIL expression *via* suppression of NF-κB pathway
	Mice	ApoE^-/-^ IL-1β^-/-^ and apoE^-/-^IL-1^+/+^ mice	Lack of IL-1β decreases the severity of AS in apoE-deficient mice
	Mice	ApoE^+/-^IL-1R1^+/-^ and apoE^+/-^IL-1R1 ^-/-^ mice	IL-1R signaling mediates atherosclerosis associated with bacterial exposure and/or HFD in a murine apoE heterozygote model
	Mice	IL-1Ra^+/+^apoE^-/-^ and IL-1Ra^+/-^ apoE^-/-^	Lack of IL-1Ra modulates plaque composition in apoE -deficient mice
	Rat	AGEs	AGEs-induced VSMCs proliferation, migration, and ECM accumulation by inducing CTGF expression *via* ERK1/2, JNK, and Egr-1 pathways
	Rat	Knockdown circDiaph3	CircDiaph3 upregulated the transcription of Igf1r and supported the proliferation and migration of VSMCs.
	Rat	Adenovirus mediated MicroRNA-195	MicroRNA-195 reduces VSMCs proliferation, migration, and prevents neointimal formation
**Migration**	Rat	HMGB1	AP-1-mediated osteoprotegerin expression in the increased migration of VSMCs stimulated with HMGB1
	Rat	Knockdown S100B by shRNA	Knockdown of S100B attenuated the PCNA expression and suppressed PDGF-BB-induced VSMCs proliferation and migration *in vitro*
**Phenotypic transformation**	Mice	Nidogen-2-/- mice	Nidogen-2 maintains the contractile phenotype of VSMCs and prevents neointima formation *via* bridging Jagged1-Notch3 signaling
	Rat	MEF2B siRNA	MEF2B-Nox1 signaling is critical for stretch-induced phenotypic modulation of VSMCs
	Rat	High phosphorus	LncRNA H19 sponges miR-103-3p to promote the high phosphorus-induced osteoblast phenotypic transition of VSMCs by upregulating Runx2
	Mice	Silencing of MFN2	Down-regulating of MFN2 promotes vascular calcification *via* regulating RAS-RAF-ERK1/2 pathway

## Diabetes and VSMCs

**Figure 1:**
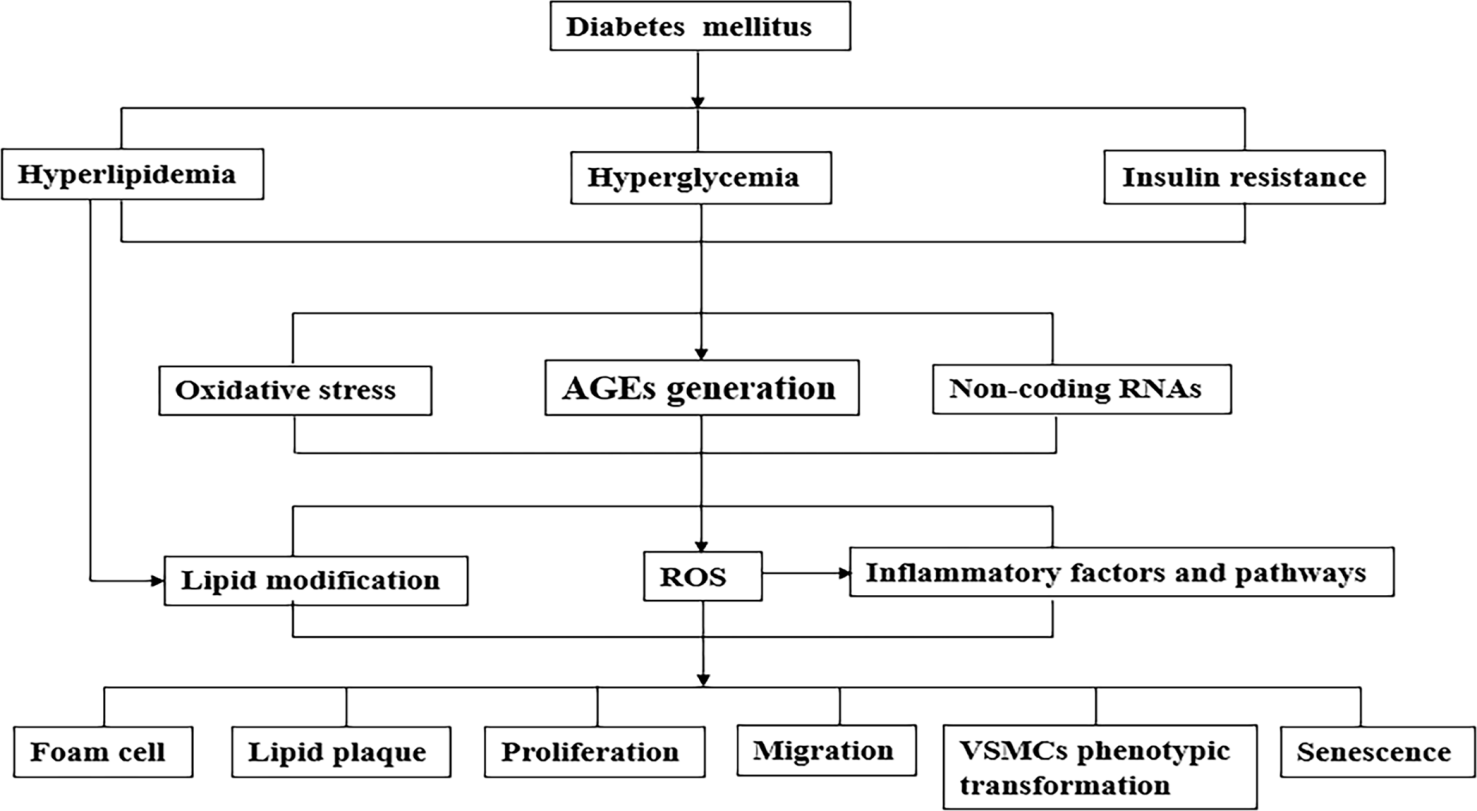
A simplified mechanism of the pathophysiological connection of the effect of diabetes on atherosclerotic VSMCs. Metabolic abnormalities (such as hyperglycemia, hyperlipidemia) and insulin resistance caused by diabetes cause a variety of pathological changes in blood vessels, including atherogenic lipoproteins, the formation of AGEs, oxidative stress and various inflammatory factors/pathways. As the above-mentioned synergistic effects of mechanisms cause phenotypic transformation of VSMCs which are functional cells of blood vessels, lead to aggravate AS development. AGEs, advanced glycation end products; VSMCs, vascular smooth muscle cells; LDL, low density lipoprotein.

The rapid increase of diabetes and its underlying patients led to a substantial increase in the number of diabetic cardiovascular complications (98). In fact, the effect of diabetes on atherosclerotic VSMCs is not simply regulated by a certain mechanism, but the result of the interaction of multiple factors (99). The simple mechanism of diabetes on atherosclerotic VSMCs was summarized in **Figure 1**. With the development of genetic engineering technology, it is possible to trace the lineage of VSMCs, study its fate map, and further study its developmental origin, plasticity, clonality and function in plaque, which provides reliable evidence for the complex role of VSMCs and VSMCs-derived cells in AS. Numerous studies demonstrate that regulating the phenotypic transformation of VSMCs and affecting their function alleviate AS severity (100–102).

In response to vascular injury, VSMCs convert from physiological contraction to pathological proliferation synthesis that through proliferation, migration, osteogenic transformation and macrophage-like transformation these play a significant role in the development and progression of AS plaque (103). The effect of glucose metabolism on VSMCs which mainly located in media is crucial. High glucose induced vasoconstriction in rat is highly sensitive that directly affects VSMCs contractile function (104). Previous studies shown that glucose concentration in diabetic patients up to 30.5 mmol/l, which can upregulate F-actin, α-SMA and cytoskeleton (105–107). Inflammatory factors such as TNF-α, IL-α, PDGF, Fibroblast growth factor 21 (FGF21) are produced at the site of high glucose injury (108), PI3K/Akt, NF-κB and other signal pathways also involved (109–113). Continuous high glucose environment in body increases the oxidation of vascular cells, making them in oxidative stress state over a long period of time, which is a key pathogenic factor for AS progression (82, 114–116). Severe vascular calcification in diabetic has also been confirmed to be related to VSMCs (117, 118). In addition, AGEs (119, 120), apoptosis (121, 122), oxidative stress (123, 124) and a variety of noncoding RNAs (125, 126) regulate AS under diabetic state. Recent studies about the effect of high glucose on VSMCs was listed in **Table 3**. Therefore, an important step for the prevention and treatment of diabetic AS is to inhibit the abnormal proliferation and migration of VSMCs.

**Table:** Table 3 Mechanisms of high glucose in VSMCs function.

Function	Subject	Treatment	Effect
Proliferation	Human	25 mM glucose+ metformin	Metformin reduced VSMCs proliferation in a concentration-dependent manner (127)
	Rat	25 mM glucose+ OA	Acarbose attenuates migration/proliferation *via* targeting microRNA-143 in vascular smooth muscle cells under diabetic conditions (128)
	Mouse	High glucose+ Pin1 or BRD4 inhibitor	Inhibition of Pin1/BRD4 pathway may improve diabetic atherosclerosis by inhibiting proliferation and migration of VSMCs (129)
Migration	Human	High glucose+ Relmβ	Relmβ augments phenotypic modulation and migration of human aortic smooth muscle cell induced by high glucose (130)
	Rat	30 mM glucose+ vitamin D	RBP4 can promote the proliferation and migration of VSMCs (131)
Calcification	Human	50 mM glucose+ 15 µM ZnSO4	zinc was found to blunt the increased expression of osteogenic and chondrogenic markers in high glucose-treated VSMCs (132)
	Rat	25 mM glucose + liraglutide	GLP-1R mediates calcification of VSMCs in diabetes patients with as by inhibiting PI3K/Akt and Erk1/2 signaling pathways (133)
	Mouse	apelin-13 or/and high glucose	Apelin-13 attenuates high glucose-induced calcification of MOVAS cells by regulating MAPKs and PI3K/AKT pathways and ROS-mediated signals (134)
Senescence	Human	30 mM glucose	LncRNA-ES3 inhibition by Bhlhe40 is involved in high glucose–induced calcification/senescence of vascular smooth muscle cells (126)
	Rat	33.3 mM glucose	Prostaglandin F2α- FP receptor ameliorates senescence of VSMCs in vascular remodeling by Src/PAI-1 signal pathway (135)

OA, oleic acid; BRD4, bromine domain protein 4; Relm β, resistin-like molecule beta.

RBP4, retinol binding protein 4; PAI-1, plasminogen activator inhibitor-1.

CREB, cAMP response element binding protein.

Cell senescence is a dynamic process of incremental development. Senescent VSMCs show a decrease in contractile protein expression. The senescent VSMCs in AS affect the size and stability of plaque (136). Senescent VSMCs secrete matrix degradation protease, which leads to collagen reduction and increases the risk of AS related complications (137). The combined treatment of Ang II and high glucose synergistically increases the proportion of aging regions in VSMCs, partly through autophagy, oxidative stress and p21-pRb pathway (138). Studies have shown that senescent VSMCs induced by proprotein invertase subtilisin/Kexin type 9 (PCSK9) is related to apoptosis pathway (139). Compared with control group, the burden of aortic plaque in diabetes mice was more serious, with fewer VSMCs, but the proportion of senescent cells in the plaque was larger (140). All above contents suggest that senescent VSMCs are closely related to the severity of atherosclerotic plaque in diabetes.

## AGEs and AS

The heterogeneous molecules of AGEs were created in the classic Maillard reaction discovered in the early 20th century (127). And more than three decades ago, a theory about aging was proposed, which hypothesized that the slow and sustained accumulation of AGEs was a causal factor in aging, and the long-term accumulation of these compounds might alter the structure and function of proteins (141, 142). This process may also lead to the pathology of metabolic diseases, such as diabetes and AS, as well as oxidative stress and inflammation associated with neurodegenerative diseases of aging (127, 143). Maillard reaction, a classical generation pathway of endogenous AGEs, which consists of three main steps: first, reducing sugars react with proteins, lipids and nucleic acids to form unstable Schiff bases through non-enzymatic reactions, then undergo structural rearrangement to form stable and irreversible complex, and finally the complex is oxidized (glucose oxidation), dehydrated and degraded to the final product (144). Under physiological conditions, slow and complex glycosylation reactions between sugars form macromolecular toxic substances AGEs (145). The production pathways of AGEs can be divided into endogenous and exogenous pathways (146). Most of the exogenous pathways are derived from the western diet which is mainly thermal processing of food, especially frying, grilling, baking or barbecuing, which are the major sources of exogenous AGEs (147). At present, more than 20 kinds of AGEs have been identified, such as carboxymethyl-lysine (CML), carboxyethyl-lysine (CEL), pentosidine and so on (148).

In addition, smoke is also another important source of exogenous AGEs, and cigarettes contain generous glycosylation products (146). In healthy population, the intake and excretion of AGEs in a dynamic balance, but pathological states such as diabetes, aging and inflammation can break the balance and accelerate the non-enzymatic glycosylation (127, 145). AGEs are metabolized by binding with corresponding receptors located on wide variety types of cells surface, which then are released by degrading into a small soluble peptide (149). Kidney is an indispensable organ in the metabolic process of AGEs. About 30% intake of dietary AGEs eliminated by kidney in healthy body (150). If kidney function is impaired, this percentage will be lower.

A growing number of studies have testified that AGEs produce a marked effect in the above-mentioned effects on the function and phenotype of VSMCs. Under the condition of hyperglycemia, AGEs formation was accelerated. Accumulated AGEs have been associated with number of diseases, including AS and diabetes (151). AGEs are a group of glycated macromolecular protein with strong resistance to protease hydrolysis that are formed irreversibly through a chain of nonenzymatic chemical reactions (152). Previous studies have shown that the levels of AGEs in diabetic patients are significantly higher than non-diabetes patients, which are bound up with the severity of vascular complications (25, 153). At the same time, the accumulation of AGEs was detected in macrophages and VSMCs of atherosclerotic vessel walls (25). The function of AGEs in AS was summarized in **Table 4**.

**Table:** Table 4 AGEs and AS.

Function	Factors	Effect
**Stiffness**	ECM, collagen	The intermolecular covalent connections or crosslinking on type-I collagen can cause molecular packing expansion, resulting in enhanced vascular stiffness (27)
**Endothelial dysfunction**	ADMA	AGEs-RAGE interaction increases oxidative stress, which can deactivate Nitric oxide and stimulate the production of dangerous peroxynitrite and ADMA which is a blocker of Nitric Oxide synthase (154)
	Proflin-1	AGEs promote endothelial hyperpermeability by triggering proflin-1, remodel and restruct of the cell actin (155)
**Oxidative stress**	Fee radical	AGEs may directly increase free radical generation by binding and activating transition metal ions (156)
	ROS	AGEs-RAGE axis stimulates pathways, including MAPK, ERK 1/2, and nuclear factor-κB (157)
**Foam cell formation**	CML, CD36	CML/CD36-driven FCs generate free cholesterol and reactive oxygen which block cell migration (158, 159)
**Calcification**	Hydrogen peroxide	Increased reactive oxygen species generation and NADPH oxidase expression in the vicinity of plaque calcification (160)
	RAGE, Galectin-3	Inflammatory cells exhibited modest amounts of RAGE and inflammatory markers, whereas VSMCs in the macro-calcified zone produced high levels of galectin-3, α-SMA, and the osteoblast development marker alkaline phosphatase (161, 162)

ADMA, a disintegrin and metalloproteinase.

## AGEs and RAGE

**Figure 2:**
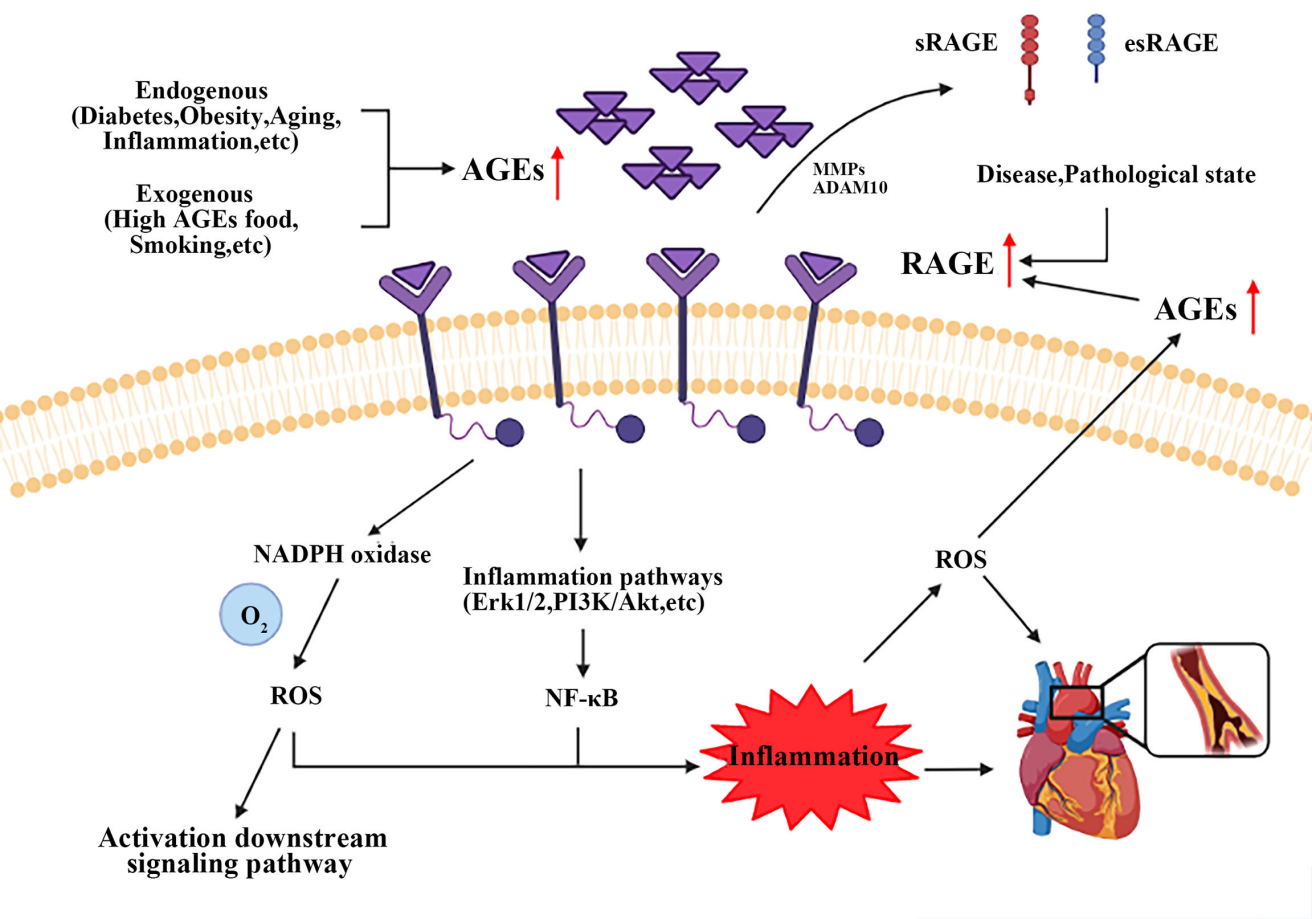
Brief mechanism of the interaction between AGEs and RAGE on VSMCs. Endogenous and exogenous pathways increase AGEs content, while disease and pathological states upregulate the expression of RAGE. AGEs-RAGE combination leads to the increase of ROS and activates inflammatory signaling pathways, such as Erk1/2, PI3K/Akt and NF-κB. All these events form a positive feedback loop, and ultimately promote AS progress. AGEs, advanced glycation end products; RAGE, receptor for advanced glycation end products. ROS, reactive oxygen species.

As described above, most notably pathogenic mechanism is the specific binding of RAGE to AGEs, see **Figure 2**. RAGE is a member of the immunoglobulin superfamily and owns multiple ligands. RAGE exists on the surface of vascular cells membrane, most in VSMCs (163). In physiological condition, the expression level of RAGE is minimal, disease can upregulate its expression to an activatable state (164). Recent studies have identified four forms of RAGE in mammals, full length cell RAGE, N-truncated RAGE, and two C-truncated RAGE which has two isoforms, cleaved RAGE (c-RAGE), and endogenous secretory RAGE (esRAGE) (165). RAGE was treated differently to form c-RAGE and esRAGE, the former was formed by proteolytic cleavage, and the latter was the product of selective mRNA splicing (166, 167). It has been found that RAGE contains complete three domains that allow ligands to function properly in their biology. sRAGE consists of c-RAGE and esRAGE (168), which are nonfunctional because neither of them do not have a complete signal transduction transmembrane domain. Both sRAGE and esRAGE can competitively bind RAGE ligands, thus antagonize RAGE mediated pathological effects (168).

## AGEs and VSMCs

**Figure 3:**
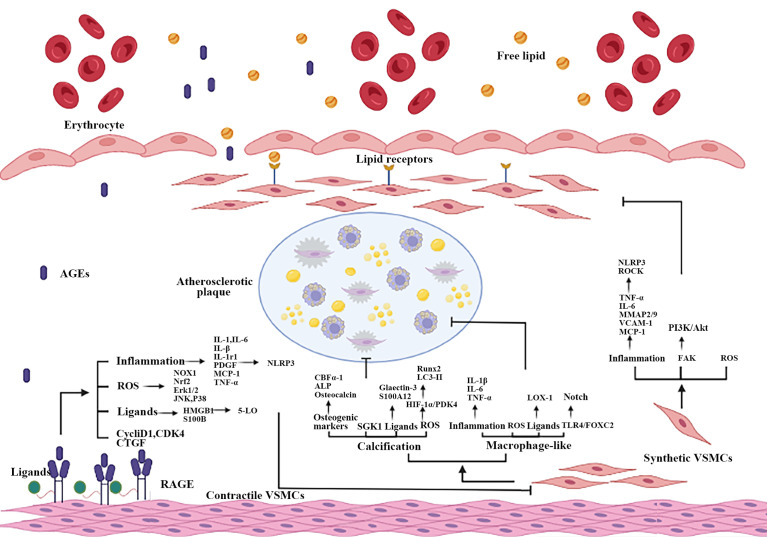
Part mechanisms of the interaction between AGEs and RAGE on atherosclerotic VSMCs. When vascular ECs are impaired, AGEs-RAGE interaction causes phenotypic transformation and VSMCs dysfunction, including proliferation, migration, calcification and the increase of macrophage-like VSMCs. Inflammation, oxidative stress, RAGE multiple ligands and other mechanisms are participated in AGEs promote atherosclerotic process by damaging VSMCs. ECs, endothelial cells.

VSMCs play important roles in AS, the contractile-synthetic phenotypic of VSMCs conversion was critical in atherosclerotic plaque formation and development (30). Abnormally AGEs (resulted from hyperglycemia) lead to the differentiation of contractile VSMCs in vessel medium into synthetic VSMCs (169). The mechanism of AGEs on VSMCs was summarized in **Figure 3**.

## Proliferation

VSMCs in the normal state are differentiated, quiescent and contracted, whereas VSMCs in the damaged state are dedifferentiated, proliferated and synthetic phenotypes (170). A growing body of evidences suggest that VSMCs exposed to AGEs exhibited a phenotype with proliferation. VSMCs proliferation has different dual roles during atherosclerotic lesion progression, VSMCs proliferation was needed in successful plaque repair and plaque stability; however, unregulated proliferation accelerates the progression of diabetic vasculopathy (171, 172). Excessive proliferation of medial VSMCs results the increased aortic stiffness and decreased aortic compliance. Blood serum levels of AGEs are elevated in CAD patients with type 2 diabetes as compared with CAD patients without diabetes (173). AGEs/RAGE interaction induced the proliferation of different species VSMCs, *via* increased ROS production and downstream pathway activation subsequently (174–176). RAGE expression was found to be significantly increased in human carotid AS (177), associated with elevated oxidative stressors locally (178). HMGB1 induces VSMCs to produce leukotrienes, increases 5-LO expression, and subsequently upregulates RAGE mRNA and protein expression in rapidly proliferating VSMCs, indirectly affecting vascular remodeling (179). Furthermore, VSMCs proliferation is suppressed in homozygous RAGE-null mice, relative to wild-type littermates. Increased ROS in VSMCs is a result of NADPH oxidase catalytic subunit differentially regulated by AGEs, which stimulates Nox1 transcription in young age, but Nox4 in aging under hyperlipidemic conditions (180). Glycated serum albumin promotes VSMCs proliferation by inducing the expression of inflammatory factors MCP-1 and IL-6 (181), which is inhibited by antioxidant N-acetylcysteine. Statins upregulate the expression of Nrf2-related antioxidant genes NQO1 and HO-1 in VSMCs through the ERK5-dependent Nrf2 pathway and inhibit the effect of AGEs-induced VSMCs proliferation (182). AGEs/ROS/ERK1/2 signaling pathway also induced VSMCs proliferation (183). Collectively, these results showed that inflammation and oxidative stress play a central role in the proliferation of VSMCs and inhibit the proliferation of VSMCs could be a useful method for AS treatment.

## Migration

In fact, VSMCs migration plays different roles in atherosclerotic plaques at different stages. In early plaques, VSMCs migration contributes to stabilize plaques through forming fibrous caps, while in late plaques, the death of migrated VSMCs accelerates plaque rupture and corresponding clinical symptoms (184). AGEs promote VSMCs migration by stimulating inflammatory cytokine production and activating multiple pathways, leading to arterial disease (161, 162). Focal adhesion kinase (FAK) is expressed selectively in VSMCs, and links extracellular matrix/integrin and growth factors, inhibited FAK/PI3K/Akt pathways could promote VSMCs migration (185, 186). It is suggested that RAGE silence by lentivirus transfection leads to reduce the phosphorylation of Akt, thus reducing the expression of migration-related proteins (187). Crocetin pretreated VSMCs downregulated TNF-α, IL-6, MMP-2 and MMP-9, at same time, significantly inhibit the migration of VSMCs and the expression of RAGE protein (188). AGEs-induced VSMCs migration is due to increased production of PDGF which mediated activation of ROCK1 *via* the JNK pathway (189). High glucose treatment significantly increased the ROS production and VSMCs migration. Cilostazol reverses these phenomena in a dose-dependent manner. The protection effect of cilostazol on AS has been considered to inhibit superoxide, resulting in attenuation of NF-κB activation, vascular cell adhesion molecule 1 (VCAM-1)/MCP-1 expression and monocyte recruitment (190). In addition, phosphorylated Erk1/2 upregulates the number of K (CA) 3.1 channels, which is a necessary condition for RAGE mediated VSMCs migration (191, 192). The migration rate of VSMCs treated with AGEs increased significantly, increasing Bcl-2-associated athanogene 3 (BAG3) and ROS are involved (193). RAGE-NADPH oxidase-ROS pathway is unique way to increase AGEs-induced expression of LCN2 (194). In diabetic apoE^−/−^ mice, activation of the ROCK1 (a branch of the TGF-β pathway) contributed to the RAGE-induced progress of AS (195).

It is reported the silencing of circWDR77 led to slower mobility in VSMCs, which was depend on miR-124 that targeting S100 calcium-binding protein A4 (S100A4) and fibroblast growth factor 2 (FGF-2) (196, 197). LncRNA-SMILR targeted miR-141 to activate RhoA/ROCK signaling, while promoting VSMCs migration (198). LncRNA LINC00281/ANXA2/NF-кB p65 signaling pathway, related to the pro-inflammatory response, promotes the phosphorylation of p65 and co-translocates with p65 into the nucleus, resulting in to VSMCs migration (199). Multiple RNAs regulate the process of AS by targeting HMGB1, a substance that plays an important role in VSMCs migration. LncRNA OIP5-AS1 regulated miR-141-3p/HMGB1 axis to promote the migration of VSMCs (200). Regulating the migration ability of VSMCs in different periods slowed the progression of AS.

## Calcification and osteogenic differentiation

For a long time, vascular calcification was considered the end stage of AS. Research results show that vascular wall calcification is not a static and random process, but an active and strictly regulated process (201). AGEs-induced VSMCs convert to osteogenic phenotype and overexpressing osteogenic markers (CBFα-1, ALP and osteocalcin) expression, resulting in to unbalance of bone metabolism and calcification of vascular wall (120, 202, 203). AGEs increased the serum- and glucocorticoid-inducible kinase 1 (SGK1) expression in VSMCs. SGK1 knockdown restrained the high glucose-induced osteogenic trans-differentiation, which required NF-κB activation (204). AGEs stimulate the secretion of inflammatory response factors and increase CBFα-1 expression through Smad signaling pathway (120). AGEs increase the expression of NADPH oxidase in apoE^-/-^ mice, induce endoplasmic reticulum oxidative stress, and promote late calcification of atherosclerotic lesions and calcification inside the aortic arch (205). One study clarified that metabolism related enzymes were closely related to ROS and VSMCs calcification (123). STZ-induced diabetes mice showed increased HMGB-1 translocation and expression, endoplasmic reticulum stress, mineralization and osteogenic gene expression (206). AGEs-treated VSMCs also exhibited a calcified phenotype (207). CML activates PDK4 to promote VSMCs calcification and glucose metabolism, which increases the expression of PDK4 by using the elevated level of ROS as a signal transduction intermediate (123). Another result showed that AGEs partially activated HIF-1α/PDK4 pathway in VSMCs to upregulate the expression level of Runx2 and aggravate the calcification of AS *in vivo* (121, 208). Organ culture of rat thoracic aorta also confirmed that AGEs promoted vascular calcification in a time-dependent manner (119). The activation of L-type calcium channels in VSMCs by AGEs is related to its osteogenic transformation, which may become a new research direction (161).

The stability of calcified plaque is related to two different AGEs receptors (galectin-3 and RAGE) (209). In carotid plaques of patients undergoing carotid endarterectomy, RAGE and inflammatory cells were only expressed in unstable inflammatory infiltrating areas with “microcalcification”, while galectin-3 and alkaline phosphatase were only expressed in fibrous areas and areas near “big calcification” (209). The targeted expression of RAGE ligand S100A12 in VSMCs isolated from apoE^-/-^ mice will aggravate the characteristics of vascular calcification and plaque instability (210). Therefore, the regulation of AGEs receptors and ligands can be used as a treatment to enhance plaque stability. The role of autophagy in VSMCs calcification is receiving increasing attention. AGEs inhibit autophagy in a time-dependent manner by simultaneously downregulating p-AMPKα and upregulating the expression of p-mTOR, increasing the osteogenic differentiation and vascular wall calcification of VSMCs (119). AGEs *via* HIF-1α/PDK4 signaling pathway increased the expression of LC3-II protein, decreased the level of p62 protein and enhanced the autophagy ability of VSMCs (121). Statins inhibit TGF-β1 by activating autophagy to induce calcification, thereby achieving vascular benefit (211). The osteogenic effect of AGEs on VSMCs increases the hardness of blood vessels and weakens the elasticity. Calcified plaques may cause adverse clinical consequences. In addition, the role of different AGEs receptors in the calcification process can provide new ideas for stabilizing calcified plaque.

## Macrophagic differentiation

The previous view considered monocytes recruited to the damaged site of blood vessels, then monocytes transformed into macrophages to phagocytize modified lipoproteins, which were the main source of foam cells in atherosclerotic plaques (212, 213). Several research results showed that VSMCs-based foam cells account for 45% to 90% approximately after exposure to lipids (such as cholesterol or lipoprotein), rather than macrophages, further highlights the important role of VSMCs in arterial disease (41, 214, 215). The level of serum CML and plaque RAGE in diabetic patients were higher than healthy people, which was significantly correlated with the number of macrophage-like VSMCs (216). AGEs downregulate VSMCs specific contractile markers (α-SMA, MYH11), decompose F-actin and increase collagen I, induce VSMCs transform to macrophage phenotype, thus losing the typical spindle appearance and lipid accumulation in VSMCs (217). Human diabetes-related atherosclerotic factors (hyperglycemia, ox-LDL, AGEs) can enhance the expression of LOX-1, suggesting that LOX-1 is involved in the interaction between CML and RAGE. LOX-1 expression has been detected in endothelial cells of early carotid AS and VSMCs of late carotid AS (114, 218). A variety of inflammatory factors (IL-1β, IL-6 and TNF-α, etc.) in the low-grade inflammatory environment of atherosclerotic vascular wall and long-term ROS stimulation accelerate the transformation from VSMCs to macrophages (219). VSMCs generate cyclic GMP in response to external stimulation and increase intravascular blood flow. Through cell fate mapping technology, PRKG1, a circulating GMP dependent protein kinase 1, is involved in the transformation of VSMCs into macrophage-like VSMCs in plaques. At the same time, the study also found that macrophage-like VSMCs came from mature VSMCs that migrated to plaque (220). VSMCs isolated from diabetes mice showed the same phenomenon (221). Indeed, whether macrophage-like VSMCs have macrophage function remains to be further studied. Weakening the effect of AGEs on promoting the transformation of VSMCs into macrophage-like VSMCs may reduce the number of foam cells in plaque.

## Conclusion

VSMCs, as the key cell of blood vessels, mainly affect AS progression through functional changes and phenotypic transformation. VSMCs dysfunction, as one of the risk factors of cardiac metabolic diseases, plays an important role in AS pathogenesis. A growing number of studies have linked AGEs to AS. However, due to the complexity and multiple factors in AGEs -induced VSMCs dysfunction, it is not yet possible to prevent or treat atherosclerosis. AGEs accelerate AS progression by promoting the main aspects of functional changes (proliferation and migration) of VSMCs. Firstly, changing lifestyle and reducing AGEs intake should be widely advocated. Secondly, high blood glucose fluctuation can promote AGEs formation. Therefore, studying the role of AGEs in AS is helpful to solve AS in diabetes and may become a therapeutic target in the future. In a word, profound study of the effect and mechanism of AGEs on VSMCs in AS should more accurately focus on the key targets and alleviate or reverse the cardiovascular complications of diabetes.

## Author contributions

LM and RY contributed equally to this work, LY and DZ contributed to the manuscript design, discussion and revision. All authors contributed to the article and approved the submitted version.

## Funding

This work was supported by grants from the National Natural Science Foundation of China (8217020350) and Beijing Natural Science Foundation (7212055).

## Conflict of interest

The authors declare that the research was conducted in the absence of any commercial or financial relationships that could be construed as a potential conflict of interest.

## Publisher’s note

All claims expressed in this article are solely those of the authors and do not necessarily represent those of their affiliated organizations, or those of the publisher, the editors and the reviewers. Any product that may be evaluated in this article, or claim that may be made by its manufacturer, is not guaranteed or endorsed by the publisher.
